# Ursolic Acid-Induced Apoptosis via Regulation of the PI3K/Akt and MAPK Signaling Pathways in Huh-7 Cells

**DOI:** 10.3390/molecules23082016

**Published:** 2018-08-13

**Authors:** Kwong-Chiu Lee, Yao-Li Chen, Ping-Yi Lin, Wan-Ling Chuang

**Affiliations:** 1 Department of Anesthesiology, Changhua Christian Hospital, Changhua 50006, Taiwan; 75102@cch.org.tw; 2 Transplant Medicine & Surgery Research Center, Changhua Christian Hospital, Changhua 50006, Taiwan; ylchen.cch@gmail.com (Y.-L.C.); 69221@cch.org.tw (P.-Y.L.); 3 Department of Surgery, Changhua Christian Hospital, Changhua 50006, Taiwan; 4 School of Medicine, Kaohsiung Medical University, Kaohsiung 80708, Taiwan

**Keywords:** huh-7, ursolic acid, apoptosis, PI3K/Akt, MAPK pathway, caspase-3, PARP

## Abstract

Ursolic acid (UA), is a kind of triterpene acid that exhibits wide biological properties. In this article, the effects of UA on apoptosis and the proliferation of human hepatoma Huh-7 cells were reported. The MTT results showed that cell viability of Huh-7 was reduced in a concentration and time-dependent effect. In addition, DAPI staining was used to detected condensation of chromatin in nucleus. Apoptotic cell population was examined using Annexin V/PI staining. The results showed that exposure to UA affected extrinsic and intrinsic pathways through, reduced expression of Bcl-2, Mcl-1, and TCTP; increased levels of the apoptotic proteins TNF-α, Fas, FADD, and Bax; and activation of cleaved caspase-3 and PARP. UA also inhibited the p-Akt and p38 MAPK signaling transduction pathways, and increased activity in the p-ERK signaling pathway. Taken together, UA inhibited the cell growth of Huh-7 cells and affected apoptosis, via regulated cellular signaling transduction.

## 1. Introduction

Hepatocellular carcinoma (HCC), is a common type of cancer in the Asia-Pacific region. HCC has a poor prognosis and high recurrence rate. Multiple factors cause HCC, such as aflatoxin B1, alcohol, drugs, and hepatitis B and hepatitis C infections [1]. HCC treatments include surgical resection, radiotherapy, liver transplantation, and chemotherapy [2]. Even though surgical resection is the most common therapy to treat HCC, the recurrence of HCC is a huge issue [3]. Therefore, molecular agents, such as Sorafenib, Brivanib, Linifanib, and Erlotinib, have been used as treatments for various cancers [4]. Sorafenib treats HCC by inhibiting the Raf protein, and then blocking the RAF/MEK/ERK signaling pathway and cell surface receptors (VEGFR-2, VEGFR-3, and PDGFR-β) [5]. The mechanism of Erlotinib, first inhibits the epidermal growth factor (EGF) signaling pathway, then causes G1/S phase cell cycle arrest [6]. The PI3K/Akt/mTOR and PPARγ pathways are significantly activated in high-grade HCC [7,8]. The MAPK pathway is an important signaling pathway in HCC [9]. According to these studies, slowing the specific signaling transduction pathway is probably a good way to treat HCC.

**Figure 1:**
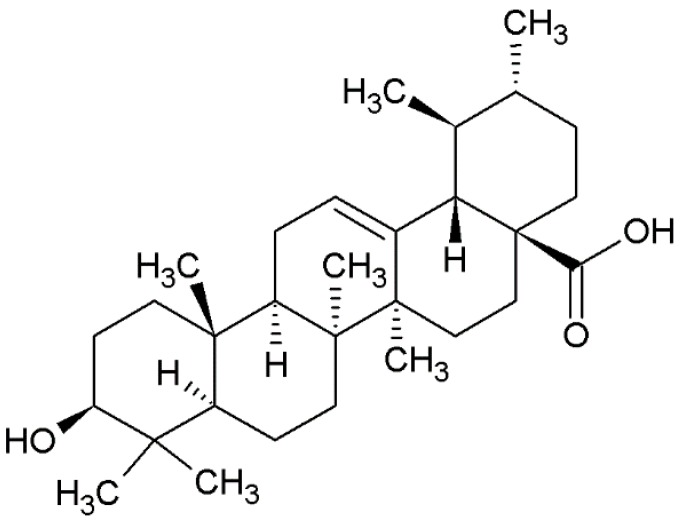
Chemical structure of ursolic acid (UA).

Ursolic acid (3β-hydroxyurs-12-en-28-oic acid) is a pentacyclin triterpenoid, containing six isoprene units (Figure 1). The molecular formula of UA is C_30_H_48_O_3_, and the structure of its α-amyrin skeleton contains two hydrogen bonds at positions 3 and 28, showing biological activities such as in position 3. The Hydroxyl group (OH) could provide a combination of a hydrogen atom and a radical; and a carbonyl group has the ability to chelate with metals [6]. UA can be found in many plants, fruits, and herbs. The biological properties of UA include anti-angiogenic activity, antibacterial activity, antiprotozoal activity, anti-inflammatory actiity, anti-viral, anti-tumor activity, and anti-oxidation [10,11,12,13,14]. Woźniak et al. reported the pharmacological activities of UA, and also evaluated its effects via clinical phase I trials [15]. Furthermore, UA has been found to affect apoptosis in numerous cancer cells. UA changes the glycolytic pathway and promotes apoptosis in breast cancer cells [16], and it inactivates the Wnt/β-catenin signaling pathway in human osteosarcoma cells [17]. UA induces apoptosis by endoplasmic reticulum stress target, to activate c–Jun N-terminal kinase signaling (JNKs) in human urinary bladder cancer T24 cells [18], and it represses the invasiveness of gastric cancer SNU-484 cells by decreasing the level of matrix metalloproteinase (MMP) [19].

The process of apoptosis changes cells morphologically, such as through membrane blebbing, cell shrinkage, cell convolution, pyknosis, karyorrhexis, chromatin condensation, and nuclear DNA fragmentation; and there is no inflammation that is different from necrosis [20]. Apoptosis plays an important role, it is essential in preserving the balance of cell death and growth. When the process is disrupted, cancer might develop [21]. Apoptosis also occurs in normal tissue development, aging cells, and the maintenance of tissue homeostasis [22].

Some findings indicated the anticancer ability of UA in many types of cancer cell lines, including the Huh-7 cells [23]. However, no studies in the literature have investigated whether UA induces apoptosis in Huh-7 cells by affecting upstream signals of the PI3K/Akt and MAPK pathways. The aim of current study is to evaluate the molecular apoptotic mechanism of UA on Huh-7 cells.

## 2. Results

### 2.1. UA Inhibits the Growth of Huh-7 Cells

**Figure 2:**
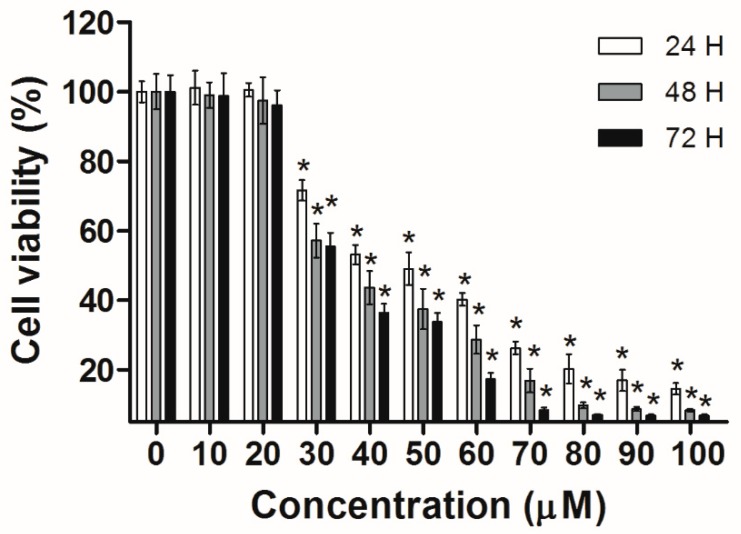
Cell viability of Huh-7 cells exposed to increasing doses of UA. The cells were seeded in plates, then treated with various doses of UA. The cell viability was assessed using the MTT. The results were presented as means ± S.D. * *p* < 0.05; compared with the untreated controls.

**Figure 3:**
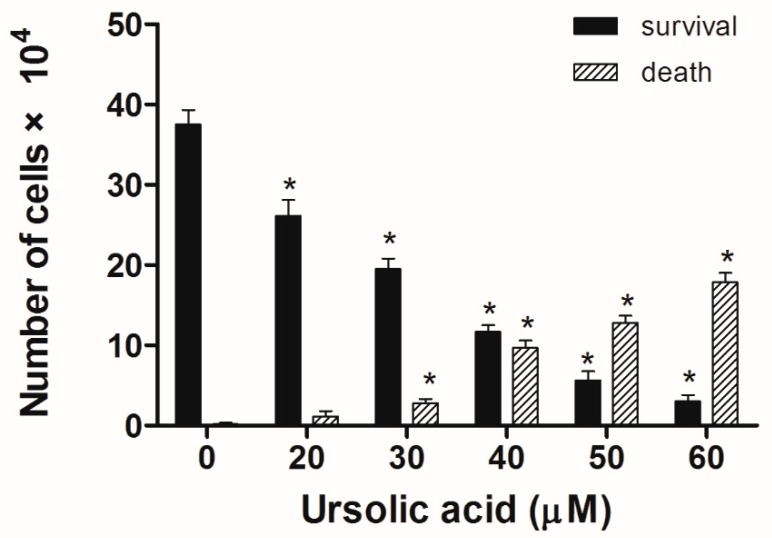
UA’s effect on cell survival and death in Huh-7 cells. The Huh-7cells were seeded in plates, then treated with different doses of UA for 24 h. Cell survival and cell death were analyzed using trypan blue staining. The survival and death of Huh-7 cells in UA was dose-dependent. The results were presented as means ± S.D. * *p* < 0.05; compared with the untreated controls.

**Figure 4:**
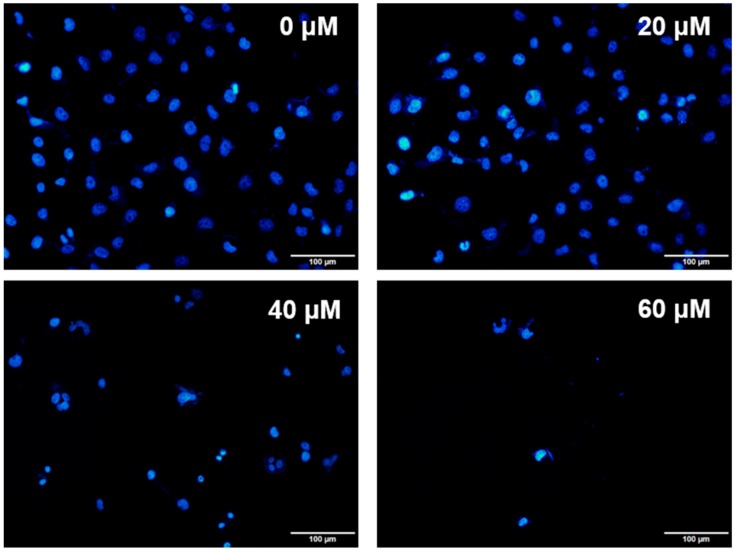
The nuclear morphology in Huh-7 by DAPI staining. Huh-7 cells after treatment with doses of UA for 24 h. Observations were taken under an Olympus IX81 microscope.

**Figure 5:**
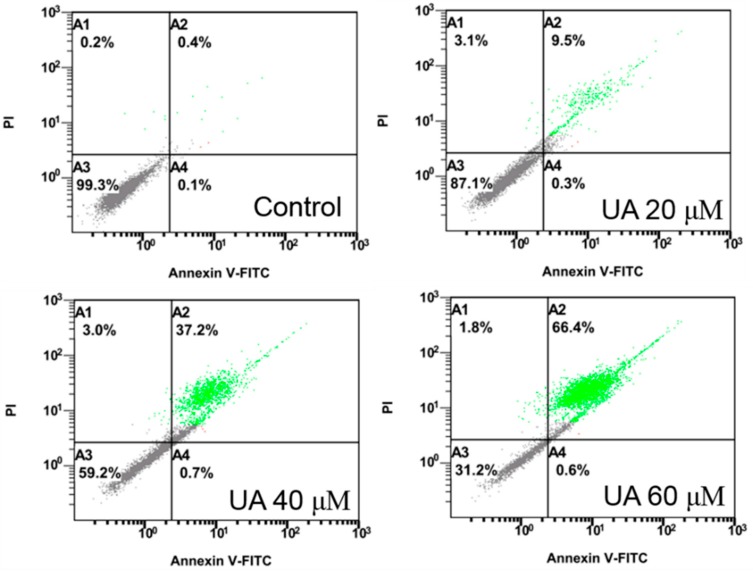
Effect of UA on apoptosis in Huh-7 cells. The cells were exposed to various doses of UA for 24 h. Annexin V/PI staining assays were used to quantify the number of apoptotic cells.

Huh-7 cells were exposed to various doses of UA at three different time intervals (24, 48, and 72 h). MTT and trypan blue assays were used to evaluate cell viability. The IC_50_ values were 53.5 ± 1.05 μM at 24 h, 45.9 ± 1.14 μM at 48 h, and 43.0 ± 0.89 μM at 72 h (Figure 2). Next, the trypan blue assay indicated that the viability decreased in a concentration-dependent manner (Figure 3). Otherwise, UA caused chromatin condensation of cell nuclei, and apoptotic bodies were observed by using DAPI staining (Figure 4). According to Figure 5, Annexin V/PI staining assay shows that the number of end stage apoptosis and dead cells (right upper section), after treatment with 20, 40, and 60 μM UA were 9.5%, 37.2%, and 66.4%, respectively. According to these findings, it demonstrated that UA suppressed the growth of Huh-7 cells in both concentration- and time-dependent manners.

### 2.2. UA Increased the Expression of Caspase-3 in Huh-7 Cells

**Figure 6:**
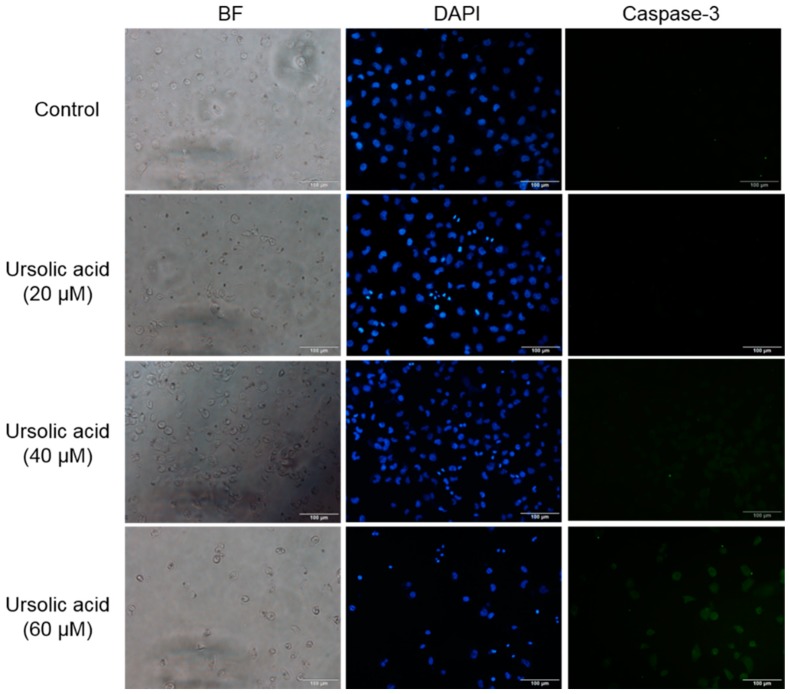
UA increased the expression of caspase-3 in Huh-7 cells. After cells were treated with various doses of UA for 24 h. The expression of caspase-3 was analyzed by immunocytochemical means.

After Huh-7 cells were exposed to different doses of UA for 24 h, the expression of caspase-3 was evaluated using immunocytochemical staining with caspase-3 antibodies. We found that UA enhanced the expression level of caspase-3 (Figure 6). Our results implied that UA may lead to apoptosis, through enhancing the level of caspase-3 in Huh-7 cells.

### 2.3. UA Induces Apoptosis in Huh-7 Cells

**Figure 7:**
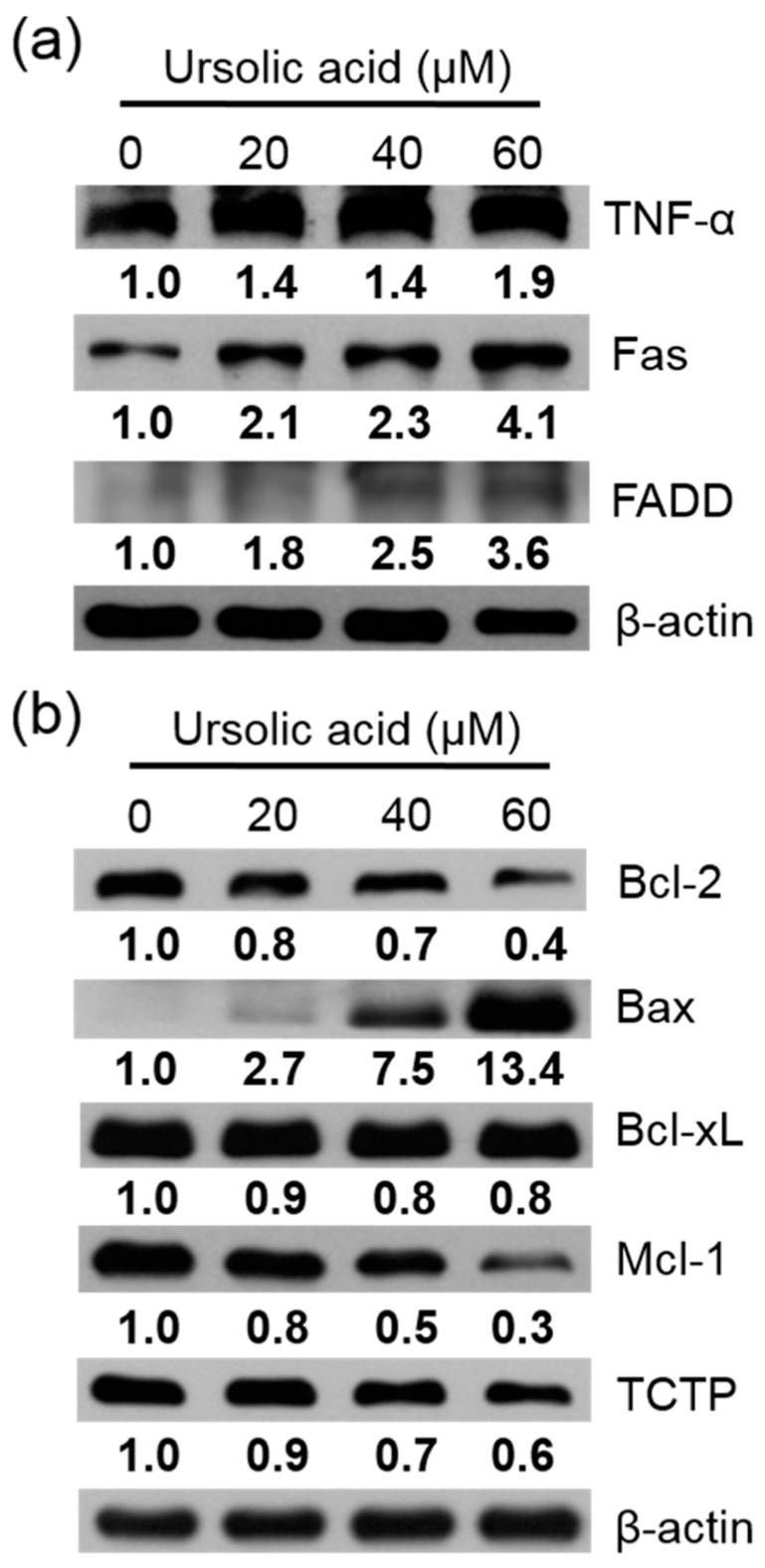
UA induced protein expression of in Huh-7 cells. (**a**) TNF-α, Fas, and FADD, (**b**) Bcl-2, Bax, Bcl-xL, Mcl-1, and TCTP. Huh-7 cells were treated with various doses of UA for 24 h. The expression of protein was evaluated using western blotting, and β-Actin was used as a loading control.

The Huh-7 cells were exposed to different doses of UA for 24 h. Then the apoptotic proteins were measured using western blotting assay. Our results showed that in cells treated with UA, the expression of TNF-α, Fas, FADD (Figure 7a), and Bax were enhanced. The expression of Bcl-2, Bcl-xL, Mcl-1, and TCTP were reduced, compared to the untreated controls (Figure 7b).

### 2.4. Caspases and PARP Expression Activated by UA

**Figure 8:**
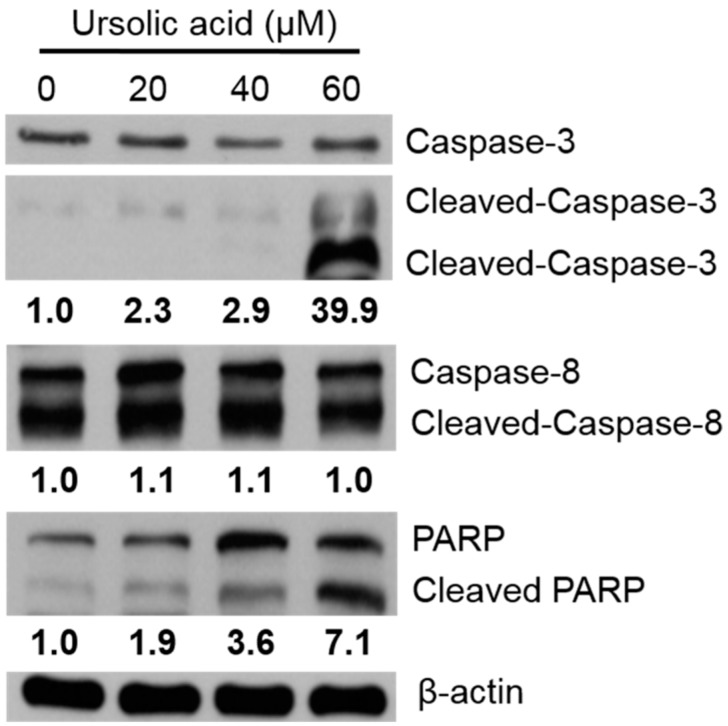
Activation of caspase-3 and PARP protein expression in Huh-7 cells. The cells were treated with various doses of UA for 24 h. The expression of protein was evaluated using western blotting, and β-Actin was used as a loading control.

Huh-7 cells were treated with various doses of UA for 24 h, and the relative expression of caspases and PARP protein were evaluated via western blotting assay. After treatment with 60 μM UA, the expression of cleaved caspase-3 significantly increased; and the expression of PARP increased after treatment with 20, 40, 60 μM UA, compared to untreated controls (Figure 8).

### 2.5. Apoptosis in Huh-7 Cells by Regulating the PI3K/Akt, p38 MAPK, ERK1/2, and JNK1/2 Signaling Pathways

**Figure 9:**
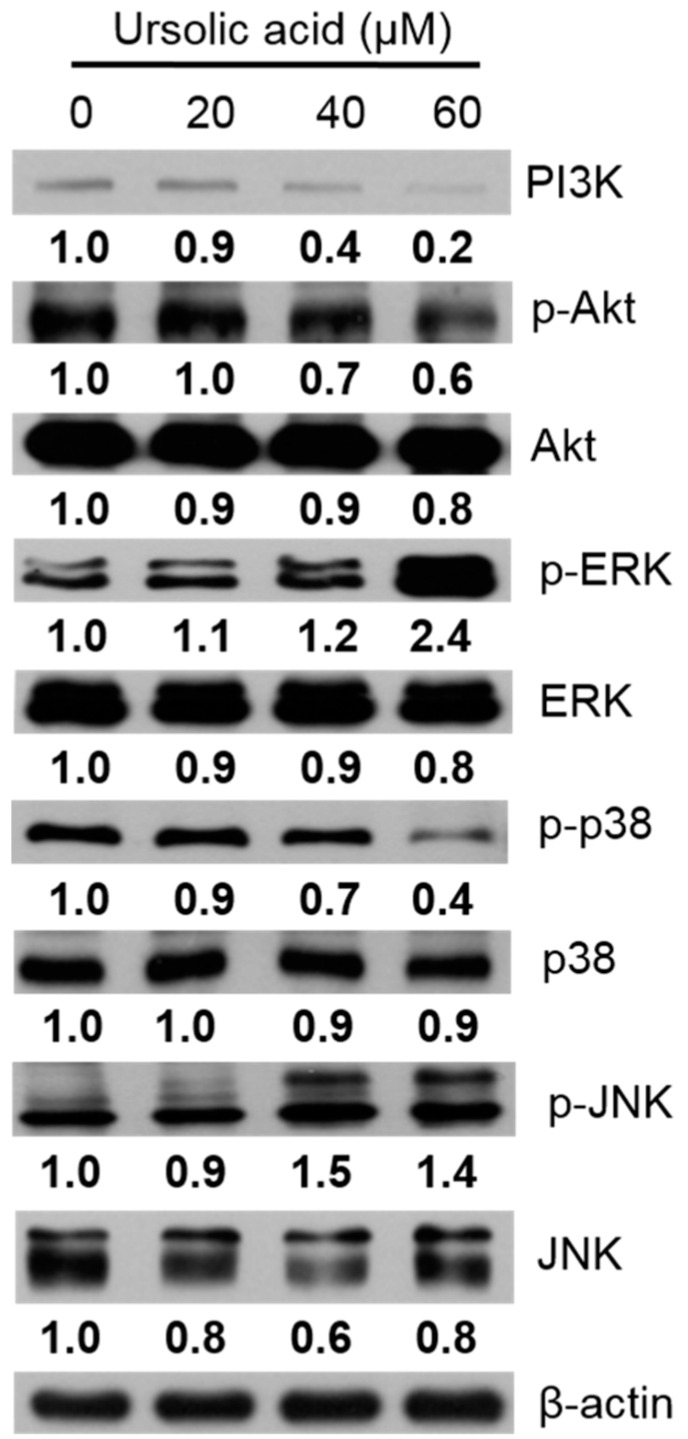
Effects of various doses of UA on the PI3K/Akt and MAPK signaling pathway in Huh-7 cells. The cells were treated with various doses of UA for 24 h. The levels of PI3K, p-Akt, Akt, p-ERK, ERK, p-p38, p38, p-JNK, and JNK were evaluated using western blotting, and β-Actin was used as a loading control.

Western blotting was used to evaluate changes in the protein expression of the PI3K, p-Akt, Akt, p-ERK, ERK, p-p38, p38, p-JNK, and JNK proteins in the Huh-7 cells, after exposure to various doses of UA for 24 h. Our results indicated the expression levels of PI3K, p-Akt, and p-p38 proteins were decreased, and the expression levels of p-ERK and p-JNK proteins were increased after treatment with UA (Figure 9).

## 3. Discussion

Apoptosis is an important mechanism in several physiological processes, including embryonic development, separation of digits, and removal of cells between digits of the upper and lower limbs via apoptosis. Cell death is essential in cardiac morphogenesis, in terms of the endocardial cushion forming a four-chambered heart [24]. Cell death also plays roles in removing excess or inappropriately connected neurons during early development of the neural system, and in balancing the immune system. B and T cells are generated and undergo apoptosis every day. Inappropriate apoptosis might induce some diseases, such as autoimmune diseases, neurodegenerative diseases, and cancer. Cancer cells develop some mechanisms to evade death and maintain survival, such as some oncogenic mutations that escape apoptosis, and the ability to disrupt the balance of anti-apoptotic and pro-apoptotic proteins. Additionally, the reduction of caspase function could cause tumor progression and metastasis. Conversely, some studies have shown that some oncogenes can promote apoptosis and induce cancer cell death; examples include, tumor cells treated with irradiation or cytotoxic agents [25].

Shyu et al. showed that apoptosis could be induced via a mitochondrial signaling pathway and command of XIAP expression in Huh-7 hepatocellular carcinoma cells; their article showed that UA induced cell viability inhibition and the IC_50_ was 75 μM [23]. Our results showed that UA can inhibit cell growth in human Huh-7 cells. UA-treated Huh-7 cells’ proliferation was inhibited in a time- and dose-dependent manner, and their nuclear morphologies were changed.

There are two major molecular mechanisms of apoptosis signaling pathways: The extrinsic pathway and the intrinsic pathway. First, the extrinsic pathway includes the tumor necrosis factor receptor-1, death receptors, CD95, the membrane-bound Fas ligand, and TRAIL receptor-1 and 2. Cell death occurs in the following manner: The ligand binding to a receptor becomes a death-inducing signaling complex (DISC), then downstream proteins such as caspase-8 and caspase-3 are activated, and they generate apoptosis. Second, in the intrinsic pathway, also called the mitochondria-associated apoptotic pathway, the Bcl-2 family proteins are the most important regulators to apoptosis. Members of the Bcl-2 family include pro-apoptotic proteins, such as Bad, Bax, Bak, Bid, Bcl-xs, and Blk; plus further anti-apoptotic proteins, such as Bcl-xL, Bcl-2, Bcl-w, and Mcl-1 [26]. UA induced apoptosis, by suppressing the level of Bcl-2 and enhancing the level of Bax proteins as reported by Woźniak et al. [15]. A previous study, showed that UA caused apoptosis by reducing the Bcl-2 expression and activating caspase-3 and PARP expression [27]. An isomer of UA-Oleanolic acid (OA), could also be an anti-cancer agent in human liver cancer cells, although UA inhibited Na^+^-K^+^-ATPase activity and accumulated more DNA fragmentation than OA [28]. In our results, UA may have induced apoptosis via regulation of Bcl-2 and Bax levels. UA also regulated extrinsic proteins (TNF-α, Fas, and FADD) and other intrinsic proteins (Bcl-xL, Mcl-1, and TCTP). The caspase family is important in apoptosis. In another study, UA induced apoptosis by activation of caspase-3, -8, and -9 in gastric BGC-803 cells and hepatocellular H22 xenograft [29]. Our study showed that UA could also activate caspase-3 and PARP in Huh-7 cells. This data showed that UA may induce apoptosis in Huh-7 cells.

The PI3K/Akt signaling pathway is a pathway that regulates cell growth and death; inhibition of PI3K and Akt expression could increase cell death [30]. The mitogen-activated protein kinases (MAPKs) family includes the ERK, SAPK/JNK, and p38 signaling pathways, which are important in the command of cellular survival, death, proliferation, and differentiation. Smolensky et al. reported doxorubicin-induced apoptosis in human bladder cells by regulation of the PI3K/Akt and MAPKs pathways [31]. UA could activate JNK, and then regulate death receptors and survival proteins to induce apoptosis [32]. Our data showed that treatment with UA, induced apoptosis in Huh-7 through inhibition of the PI3K/Akt and p38 pathways and activation of the ERK1/2 and JNK pathways.

## 4. Materials and Methods

### 4.1. Reagents and Antibodies

UA (Santa Cruz, CA, USA) was over 98% purity, and its chemical structure is presented in Figure 1. MTT (3-[4,5-dimethylthiazol-2-y1]-2,5-diphenyltetrazolium bromide) was purchased from Merck (Darmstadt, Germany). BSA (Bovin Serum Albumin), Triton^TM^ X-100, Paraformaldehyde, Trypan blue solution, and β-actin were purchased from Sigma-Aldrich (St. Louis, MO, USA). FBS (Fetal bovine serum), DMEM (Dulbecco’s Modified Eagle Medium), penicillin-streptomycin, and PBS (phosphate-buffered saline) were purchased from Gibco BRL (Grand Island, NY, USA); and DAPI (4′6-diamidino-2-phenylindole) was purchased from Invitrogen (Carlsbad, CA, USA). Pierce™ BCA Protein Kit was purchased from Thermo Fisher (Waltham, MA, USA). Western Bright^TM^ Quantum HRP substrate solution was purchased from Advansta (Menlo Park, CA, USA). Tween-20 was purchased from Amresco (St. Louis, MO, USA); and RIPA lysis and extraction buffer was purchased from Millipore (Billerica, MA, USA). Antibodies to Fas, Bcl-2, and caspase-8 were purchased from Novus Biologicals (Littleton, CO, USA). Antibodies to TNF-α, FADD, Bax, Bcl-xL, Mcl-1, TCTP, caspase-3, PARP, PI3K, phospho-Akt, Akt, phospho-ERK1/2, ERK1/2, phospho-p38, p38, phospho-SAPK/JNK, and SAPK/JNK were purchased from Cell Signaling (Beverly, MA, USA).

### 4.2. Cell Line

Human liver carcinoma Huh-7 cells were purchased from the Bioresource Collection and Research Center (Hsinchu, Taiwan), and were cultured in 90% DMEM medium supplemented with 10% FBS, 1% penicillin, 1% streptomycin, 1% sodium pyruvate, and 1% nonessential amino acid in a humidified and 5% CO_2_ environment at 37 °C.

### 4.3. Cell Cytotoxicity

Viability of cells was measured using MTT. Huh-7 cells were seeded in a 96-well with 2 × 10^4^ cells per well. The various doses (10, 20, 30, 40, 50, 60, 70, 80, 90, and 100 μM) of UA were added to the cells. After 24, 48, or 72 h, MTT (the final concentration of 1 mg/mL) was added to each well and incubated for 2 h. We removed the medium and used 100 μL of DMSO to dissolve the dark blue formazan precipitate, and absorbance was assessed at 570 nm using a microtiter plate reader (Thermo Fisher Scientific, Waltham, MA, USA). Results were presented as percentages of MTT reduction, compared with the untreated controls.

### 4.4. Trypan Blue Assay

The Huh-7 cells were plated on a six-well with 2 × 10^5^ cells per well and allowed to attach. After treatment with different doses (0, 20, 30, 40, 50, and 60 μM) of UA for 24 h, the cells were trypsinized, centrifuged, and stained with trypan blue. Then they were transferred to a hemocytometer, which counted the viable and dead cells. Our results, were presented as percentages of the untreated controls.

### 4.5. Immunocytochemical Staining

Huh-7 cells were plated on a six-well, then treated with different doses 20, 40, and 60 μM of UA for 24 h, we then removed the medium and drugs and washed the cells with PBS. Afterwards, the cells were fixed in 4% paraformaldehyde for 30 min, then permeabilized with 0.25% Triton X-100 for 5 min on the ice. After that, we washed the cells twice with PBS, and then incubated them in 0.1% BSA for 1 h at room temperature (RT). After that, the cells were separately incubated with primary antibody anti-caspase 3 (1:200) overnight at 4 °C. Thereafter, they were washed three times with PBS and incubated in secondary fluorescent conjugated antibody (1:500) for 1 h. After being washed in used PBS three times, the cells were then incubated with DAPI (1 μg/mL) for 10 min. After being rinsed with PBS, the cells were immediately observed using an Olympus IX81 microscope (Olympus, Tokyo, Japan).

### 4.6. Annexin V/PI Double Staining Assay

We used Annexin V/FITC to detect apoptotic cells. After UA treatment with Huh-7 cells, cells were collected and then rinsed with PBS. Cells were re-suspended in 100 μL binding buffer, and then 5 μL of Annexin V-FITC and 10 μL of PI were added for 15 min at RT in the dark. Thereafter, 500 μL of binding buffer was added. Then the samples were examined using a Cytomics^TM^ FC500 flow cytometer (Beckman Coulter, FL, USA).

### 4.7. Cell Lysis and Immunoblot Analysis

Huh-7 cells were incubated for 24 h with UA, the cells were rinsed with PBS and the cells were scraped from the dishes. The proteins of Huh-7 were lysed in extract buffer with protease inhibitors. The proteins were centrifuged for 10 min at 13,000× *g*. Protein concentrations were analyzed using a BCA protein assay kit. Equal amounts of protein samples were separated into SDS-PAGE on 8%–12% polyacrylamide gel, then transferred onto PVDF (polyvinylidene difluoride membranes). The blots were blocking in PBS with 0.1% BSA and 0.05% tween 20 for 1 h, at RT. Then the membranes were incubated with primary antibodies overnight, rinsed with PBST, and then incubated with secondary antibodies conjugated with horseradish peroxidase. Membranes were rinsed three times with PBST buffer. The membranes were visualized by incubating the membranes in ECL reagent and were exposed to super RX-N film (Fujifilm Corporation, Tokyo, Japan).

### 4.8. Statistical Analyses

Statistical analyses were performed by using GraphPad Prism software, version 4.0 (GraphPad Software, Inc., La Jolla, CA, USA). All quantitative data were presented as the mean ± standard deviation (S.D.). Statistical analysis two-group comparisons were conducted using the Student’s *t*-test. A *p*-value less than 0.05 was considered to indicate statistical significance.

## 5. Conclusions

Taken together, this article is the first to demonstrate that UA could induce apoptosis in human Huh-7 cells through regulating the PI3K/Akt, p38, ERK, and JNK pathways. These findings suggest that UA can be a potential agent for anticancer activity in Huh-7 cells.

## Abbreviations

EGF	epidermal growth factor
PI3K	phosphoinositide 3-kinase
MAPK	mitogen-activated protein kinase
UA	ursolic acid
p38	p38 MAP kinase
ERK	extracellular signal-regulated kinase
JNK	cJun N-terminal kinase
PARP	poly (ADP-ribose) polymerase
TNF-α	tumor necrosis factor alpha
Bcl-2	B-cell lymphoma 2
TCTP	translationally controlled tumor protein
